# How do host age and nutrition affect density regulation of obligate versus facultative bacterial symbionts? Insights from the tsetse fly

**DOI:** 10.1093/ismeco/ycaf108

**Published:** 2025-06-27

**Authors:** Mathilda Whittle, Antoine M G Barreaux, Lee R Haines, Michael B Bonsall, Sinead English, Fleur Ponton

**Affiliations:** Faculty of Life Sciences, University of Bristol, Bristol, BS8 1TQ, United Kingdom; School of Natural Sciences, Macquarie University, Sydney, 2109, Australia; UMR INTERTRYP, Département BIOS, CIRAD, Montpellier, 34398, France; Animal Health Theme, ICIPE, Nairobi, 30772-00100, Kenya; Department of Biological Sciences, University of Notre Dame, Indiana, 46556, United States; Department of Biology, University of Oxford, Oxford, OX1 3SZ, United Kingdom; St Peter’s College, Oxford, OX1 2DL, United Kingdom; Faculty of Life Sciences, University of Bristol, Bristol, BS8 1TQ, United Kingdom; School of Natural Sciences, Macquarie University, Sydney, 2109, Australia

**Keywords:** evolutionary history, facultative, host diet, host regulation, obligate, symbiont density

## Abstract

Host–symbiont relationships can vary tremendously in the extent to which hosts depend on and control their symbionts. Obligate symbionts that provide micronutrients to their host are often compartmentalised to specialised host organs and depend on their hosts for survival, whereas facultative symbionts retain the ability to survive outside of their hosts. Few studies compare the extent to which a host controls and adjusts the density of obligate and facultative symbionts directly. We used tsetse as a model for teasing apart the relationships between a host (*Glossina morsitans morsitans*) and obligate (*Wigglesworthia glossinidia*) and facultative (*Sodalis glossinidius*) symbionts. We hypothesised that tsetse actively regulate the density of *Wigglesworthia* according to the host’s requirements, depending on their current nutritional state and developmental age. In contrast, we postulated that *Sodalis* retains some independence from host control and that the growth of this symbiont is dependent on the conditions of the immediate environment, such as nutrient availability. Using qPCR, we examined how symbiont densities change across host age and the hunger cycle. Additionally, we investigated how host nutrition influences symbiont density, by comparing tsetse that were fed nutrient-poor or vitamin enriched diets. We found that the density of *Wigglesworthia* was not influenced by the nutritional status of the host but reflected long-term host nutritional needs. In contrast, the density of facultative *Sodalis* depended on the nutrient availability. We propose that tsetse tightly regulate *Wigglesworthia* but exert only partial control over *Sodalis* growth due to the relatively recent transition of this symbiont to host-associated living.

## Introduction

Many eukaryotic organisms, such as animals, plants, and protists, live with symbiotic microorganisms within their bodies [1–3]. A great diversity exists in the nature of such symbioses. On one end of the spectrum, parasitic symbionts exploit the host for their own benefit, while on the other end, mutualistic symbionts provide benefits that increase host fitness [4]. There are many examples of mutualistic associations between microbes and hosts, such as photosynthetic algae providing essential nutrients to cnidarian hosts [5], protective bacteria in nematodes producing chemicals to defend their host from pathogens [6], and symbionts improving their insect hosts’ tolerance to heat stress [7]. Some mutualisms can evolve to become obligate for both the host and the symbiont (i.e. the host depends on the symbiont for survival and vice versa [8]).

Supporting a symbiont population, even an obligate one, entails a metabolic cost for the host because symbionts acquire all their nutritional resources from within the host [9]. The net benefit (i.e. the difference between the benefit provided to the host and the cost of supporting the symbiont) for a host participating in a mutualistic symbiosis depends on the ecological context [10–17]. For instance, the diet of many insects is supplemented with micronutrients produced by gut symbionts [18, 19], and the associated benefit depends on the host’s nutritional requirements and the availability of such micronutrients in the host’s diet. As the size of the symbiont population within the host tissues likely correlates with the amount of nutrients provisioned to the host, as well as the metabolic cost of maintaining the symbiont, the active host regulation of symbiont densities according to the host requirements, and the availability of dietary nutrients could allow hosts to maximise the net benefits [20]. Empirical studies suggest that symbiont density regulation according to host requirements occurs in several obligate nutritional symbioses of insects (reviewed in [21]). Several examples of this are: aphids harbour different symbiont densities according to their host plant [13], weevils remove their symbiont after maturation [16], and female tsetse flies (which have comparatively large reproductive investment [22]) host higher symbiont densities than the males [11, 23].

Many obligate symbioses of insects are ancient associations [24]. Long coevolutionary histories between hosts and symbionts have resulted in highly reduced symbiont genomes, which limit the ability of obligate symbionts to survive outside hosts and regulate their own replication [25]. In contrast to obligate symbionts, facultative symbionts, although potentially beneficial, are not strictly required by the host for survival and often represent more recent transitions to host-associated living [26]. Many facultative symbionts have retained the ability to survive outside of host tissues, likely due to lower gene erosion, and demonstrate a more extensive tissue distribution in the host body [27]. Several adaptations that grant a host control over its symbioses have been proposed [21]. These include compartmentalisation of symbionts into specialised housing cells and organs [28], immune responses that restrict symbiont proliferation and tissue invasion [29], and, potentially, the ability to regulate symbiont transcription in order to control symbiont metabolism and nutrient production [30].

Here, we investigate the degree of control simultaneously exerted by a host over the density of an obligate and a facultative symbiont. One issue of using symbiont density to infer host control of a symbiosis lies in attributing changes in density to host-mediated regulation (as opposed to any direct effects of the ecological context on bacteria growth). By comparing two symbionts of the same host with different evolutionary histories, under several ecological scenarios, we aimed to provide a broad set of evidence for the potential influence of host control on symbiont density. We hypothesised that while the host may maintain and adjust the density of its obligate symbiont according to its requirements, the facultative symbiont retains a greater degree of independence from the control of its host. As such, growth of the facultative symbiont is likely to be more directly affected by the immediate environment (i.e. the nutrient availability within host tissues).

We tested our hypotheses using tsetse (*Glossina* spp.), and their association with obligate (*Wigglesworthia glossinidia*) and facultative (*Sodalis glossinidius*) symbionts, as a model system. Tsetse are exclusive blood feeders and rely on *Wigglesworthia* for crucial B vitamins not available in sufficient concentrations in their diet [31]. Having coevolved with tsetse for 50–80 Ma [32], *Wigglesworthia* has achieved a highly integrated role in tsetse biology as characterised by an extremely reduced genome (0.7 Mb) [33, 34] and mutually obligate status with all *Glossina* hosts [32, 35]. The primary population of *Wigglesworthia* is located intracellularly, within the specialised organ known as the bacteriome (mycetome), which saddles the anterior midgut [31]. Female tsetse, unlike most flies, do not lay eggs but produce one offspring at a time. The single larva is fed on a milk-like substance *in utero* [36], and a secondary population of *Wigglesworthia* exists within the milk glands, which allows the maternal transmission of this symbiont to tsetse offspring [37]. Compartmentalisation of symbionts to specialised housing organs, such as the bacteriome or milk glands, helps a host control its symbiont populations both in maternal and offspring generations [28], and as such, tsetse may have the ability to tightly regulate the abundance of *Wigglesworthia.*

The tsetse secondary bacterial symbiont, *S. glossinidius*, is widespread throughout insectary and field tsetse populations and represents a relatively recent transition from free-living bacteria to an endosymbiotic lifestyle [38]. Accordingly, *Sodalis* can colonise multiple tsetse tissues including the hemolymph, midgut, fat body, milk glands (in females, where it is transmitted to host offspring alongside *Wigglesworthia* [37]), and testes and spermatophore (in males, where it can be transmitted horizontally during mating [39]). Within tsetse tissues, *Sodalis* demonstrates a dependency on thiamine (vitamin B_1_) produced by *Wigglesworthia* as it lacks the ability to produce this vitamin itself [40]. However, *Sodalis* retains the ability to survive and grow outside of host tissues in *in vitro* cultures [41]. How tsetse benefit from *Sodalis* is uncertain; although the bacteria is not found in all tsetse populations, antibiotic removal of *Sodalis* from tsetse appears to result in decreased longevity and reduced susceptibility of the host to trypanosome infection [42].

**Figure 1:**
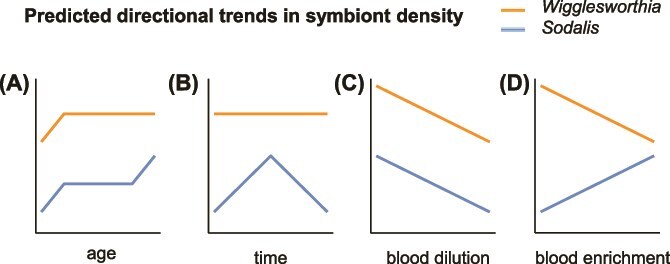
Predicted directional trends in the density of Wigglesworthia and Sodalis: (A) Study 1: across adult female age, (B) Study 2: over 96 hours following a blood meal, (C) Study 3: from hosts reared on diets of diluted blood, and (D) Study 3: from hosts reared on diets of blood enriched with yeast extract. The lines indicate trends in symbiont density rather than the densities of *Wigglesworthia* and *Sodalis* relative to each other.

Here, we used quantitative PCR (qPCR) to measure the density of *Wigglesworthia* and *Sodalis* genomes associated with the digestive and reproductive tissues of tsetse, under several ecological scenarios. First, we measured symbiont density at multiple time points across adult female age. Following an initial proliferation in early adulthood (as is observed of symbionts of several species, including tsetse [10, 17, 43–47]), we predicted that *Wigglesworthia* is maintained at a constant level to support the nutritional demands of reproduction (Fig. 1A). As thiamine provisioning from *Wigglesworthia* increases, we predicted that the density of *Sodalis* would increase in parallel to *Wigglesworthia* [17]. Additionally, maturation and senescence of the host immune system [48, 49] may reduce the ability of tsetse to control the density of *Sodalis* in early adulthood and ageing hosts, respectively, thus proliferation may be observed at these periods (Fig. 1A). Second, we measured symbiont density following a blood meal. We predicted that, due to its extracellular localisation in the midgut, *Sodalis* may demonstrate brief proliferation in the nutrient-rich environment, but tsetse will maintain a constant density of *Wigglesworthia* (Fig. 1B) according to the host’s long-term requirements for B vitamins. Third, we measured symbiont density across different diets: either diluted blood with reduced nutritional content or blood supplemented with yeast extract thereby enriched with B vitamins (as in [50]). Where energy is limiting, supporting a symbiont population could become relatively more costly to the host, and so we predicted that the density of *Wigglesworthia* will be lower in hosts receiving nutritionally poor blood (Fig. 1C). *Sodalis* may also grow to lower densities in hosts receiving poorer diets (Fig. 1C) due to fewer resources being available within host tissues. Providing an exogenous source of B vitamins undermines the benefit *Wigglesworthia* provides to tsetse; therefore, we predicted that the density of this symbiont will be actively reduced by hosts receiving an enriched diet as it is surplus to requirements (Fig. 1D). In contrast, we predicted that *Sodalis* may proliferate in the presence of a nutritionally enriched environment (i.e. containing macronutrients and thiamine from yeast extract) (Fig. 1D).

## Materials and methods

### Tsetse rearing

We obtained *Glossina morsitans morsitans* from the Liverpool School of Tropical Medicine—either at the adult stage (Study 1: female age) or as pupae (Study 2: male hunger cycle; Study 3: female dietary manipulation experiments). Individuals were maintained at 25°C with 70% humidity, with 12:12-hour light:dark cycles. Flies were fed on sterile defibrinated horse blood (TCS Biosciences, UK) three times a week (Monday, Wednesday, and Friday) using an artificial membrane system (see supplementary information for more detail). Pupae were incubated under the same environmental conditions until emergence.

### Measuring symbiont density dynamics across adult age, hunger cycle, and host diet

To investigate symbiont density dynamics across host age, we used females from 16 different age groups spanning the age range of the tsetse colony; from ~6 hours to 88 days post emergence, with *n* = 9 or 10 for each group. To investigate symbiont density dynamics across the hunger cycle, we starved adult males (at 4 weeks old) for 4 days prior to the final meal, which were then killed and processed 6 (*n* = 13), 24 (*n* = 14), 48 (*n* = 12), or 96 (*n* = 13) hours following the final meal.

To investigate the effects of host diet on symbiont density, we reared females for 6 weeks from emergence on one of 10 dietary treatments (Table 1). We used normal saline (0.9% NaCl (w/v) in water) to reduce the nutritional content of the diet at varying ratios and yeast extract to fortify the diet with B vitamins at increasing concentrations. Previous work has shown that diluting blood causes nutritional stress, i.e. flies could not compensate even if they take an increased volume of blood meal [51]. We used yeast extract as it is relatively stable in solution compared to purified B vitamins. Yeast extract and other yeast derivatives have been used to supplement the diet of tsetse in previous studies, usually without adverse effects on the flies [52, 53].

**Table 1:** Treatment groups for dietary manipulation (Study 3).

Group	Treatment	Sample size at start	Sample size for qPCR
Control	Standard defibrinated horse blood	15	12
Blood diluted with normal saline	3:1 v/v blood:saline	15	13
	2:1 v/v blood:saline	15	13
	1:1 v/v blood:saline	15	10
	0.5:1 v/v blood:saline	15	12
	0.3:1 v/v blood:saline	15	8
Blood supplemented with yeast extract	0.5% w/v yeast extract	10	9
	1% w/v yeast extract	10	9
	2% w/v yeast extract	10	9
	5% w/v yeast extract	10	3

Flies were reared for six weeks (from emergence) on their respective treatments. Surviving flies were processed for qPCR analysis.

**Figure 2:**
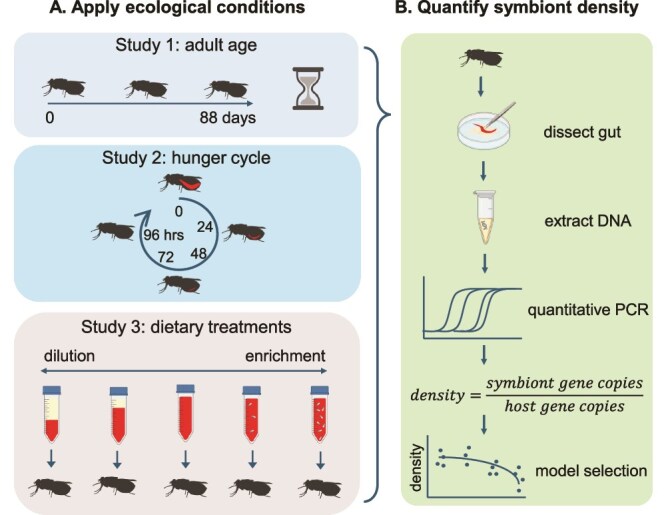
Experimental workflow. Symbiont density was measured in tsetse from three different ecological scenarios: across adult age, at various points during the hunger cycle, and subjected to diluted or enriched dietary treatments. We removed the abdominal content of tsetse bodies, including the digestive and reproductive tissue, then extracted the total DNA from each individual. We used qPCR with species-specific primers to target single-copy genes for *Glossina*, *Wigglesworthia*, and *Sodalis*. Symbiont density was calculated as the ratio of symbiont genomes to host genomes. We then used AIC model selection to analyse the emergent trends in symbiont density. Created in BioRender. lab, K. (2025) https://BioRender.com/7t274nu

To ascertain any detrimental effects of the treatments on host survival or reproduction, we counted the number of living females daily and collected any pupae produced by each treatment group. We recorded pupal weight at deposition, and for those that emerged successfully, the number of days for the unfed adult offspring to die post emergence. All surviving females were killed and processed 48 hours after their final blood meal. See Fig. 2 for a schematic of the experimental workflow, and the supplementary information for a detailed protocol for each study.

### Sample preparation and qPCR quantification of symbiont density

We killed all flies by freezing at −80°C prior to processing for qPCR quantification of symbiont density and measured the length of the “hatchet” wing cell as a standard measure for host size [54]. We removed the total content of the abdomen by dissection, including the digestive tract, reproductive tissues, fat bodies, and Malpighian tubules (Fig. 2). We then estimated the stage of pregnancy (i.e. egg or first, second, or third larval instar) for females [55] and removed any second and third instar larvae from the samples. Total genomic DNA of the remaining abdominal content was extracted using the DNeasy blood and tissue kit (Qiagen, CA), following a modified version of the manufacturer’s protocol (see supplementary information for more detail).

We used pairs of specific primers (for PCR and qPCR amplification) for species-specific and single-copy genes of *G. morsitans morsitans* (*alpha-tubulin*, GenBank Accession no. ADD19945.1), *W. glossinidia* (thiamine biosynthesis protein (*thiC*), GenBank Accession no. AGG38086.1) and *S. glossinidius* (*exochitinase*, GenBank accession no. BAE74749.1) (Table S1).

Quantitative (real time) PCR amplification was performed using a CFX384 Touch Real-Time PCR Detection System (Bio-Rad, CA) for each primer pair (see supplementary information for cycling conditions and assay optimisation protocol). Amplifications were obtained using PowerUP SYBR Green master mix (Applied Biosystems, MA). We performed each assay in triplicate, including negative controls containing no template.

We included standard curves on each qPCR plate for absolute quantification of the target genes (Fig. 2; Table S2). The C_q_ for each sample was used to estimate the quantity of target DNA (in nanograms) initially present in the template by comparing against the standard curve. We then calculated the number of gene copies for each primer pair according to Equation (1) [47], where the amplicon size is given by the number of base pairs (Table S1). As symbiotic bacteria, including *Wigglesworthia* and *Sodalis*, can demonstrate polyploidy [17, 56], we defined the symbiont density as the number of symbiont genomes (rather than the number of symbiont cells) divided by the number of *G. morsitans* genomes (as per methodology of [17, 47, 58]). As symbiont cells can demonstrate gigantism [57] and the relative cost and benefit of hosting a symbiont is likely to scale with the abundance of symbiont genomes, we concluded that this was an appropriate measure of symbiont density.


(1) \begin{equation*} Number\ of\ genomes=\frac{quantity\ of\ DNA\ target\ in\ ng\times 6.022\times{10}^{23}\ }{amplicon\ size\times{10}^9\times 650} \end{equation*}

### Statistical analysis

**Figure 3:**
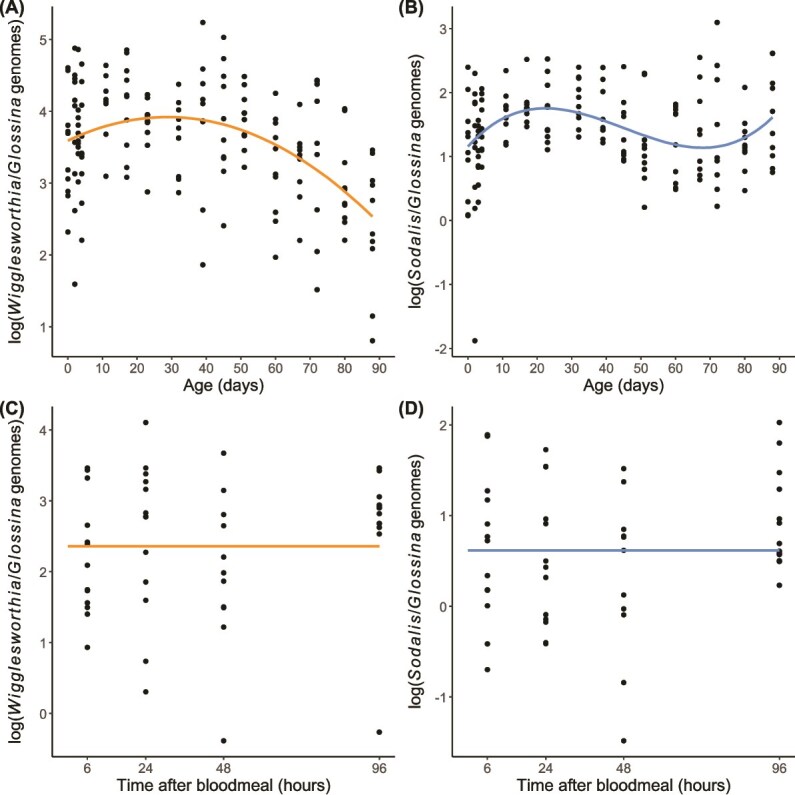
Density dynamics of *Wigglesworthia* (orange) and *Sodalis* (blue) (A–B) throughout adult development (females) and (C–D) throughout the hunger cycle (4-week-old males). Regression lines indicate predictor effects of selected models (Tables S3 and S4). Note that the *y*-axis is on different scales between the two symbionts.

**Figure 4:**
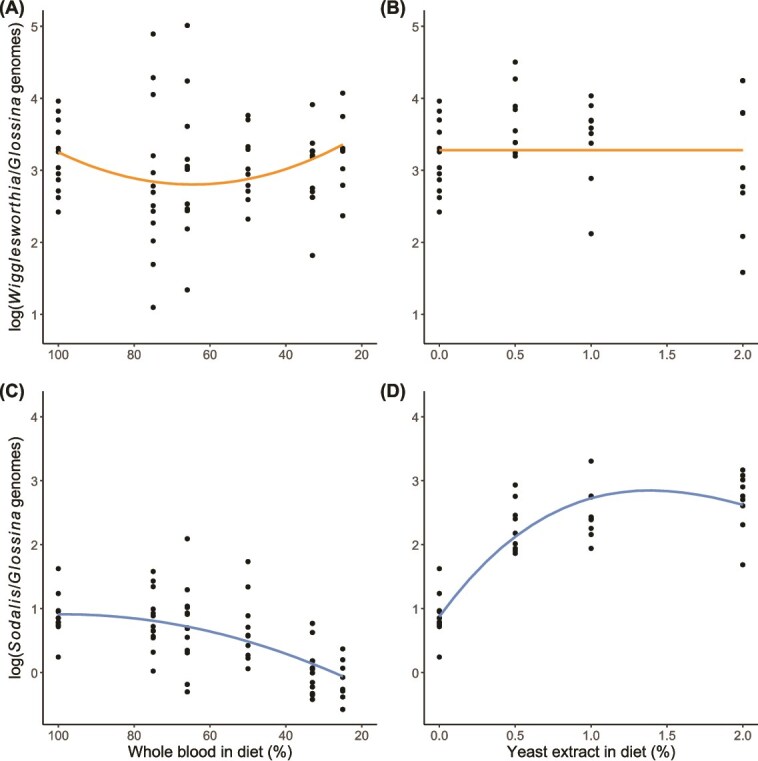
Effect of dietary manipulation on the density of *Wigglesworthia* (orange) and *Sodalis* (blue) in 6-week-old female tsetse. (A) *Wigglesworthia* density according to blood concentration (%). (B) *Wigglesworthia* density upon yeast extract (% w/v) supplementation. (C) *Sodalis* density according to blood concentration (%). (D) *Sodalis* density upon yeast extract (% w/v) supplementation. Regression lines indicate predictor effects of selected models (Table S6). Note that the *y*-axis is on different scales between the two symbionts.

**Figure 5:**
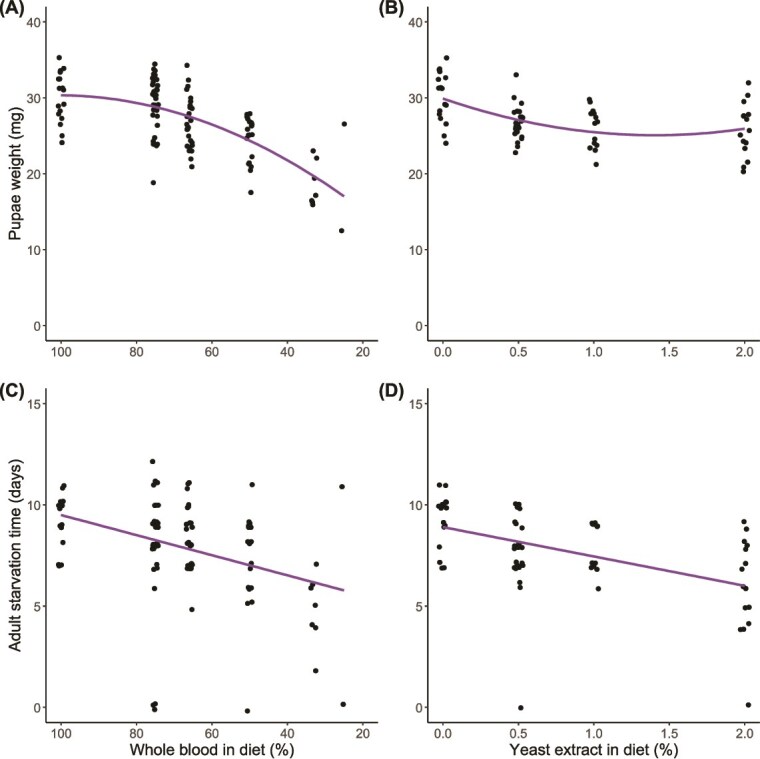
Effect of blood dilution with saline and blood enrichment with yeast extract on host reproductive output. (A–B) Weight of pupae at time of deposition. (C–D) Time taken for unfed offspring to starve upon emergence. Lines indicate predictor effects of selected models (Table S7).

The scale at which we seek to make inferences are given in Table S3. We analysed data using R (version 4.2.1) [58]. Density data were log transformed to satisfy normality assumptions and analysed using multiple linear regression. To determine if the *Wigglesworthia* and *Sodalis* density data corresponded qualitatively to the trends we predicted (Fig. 1), we used Akaike’s information criterion (corrected for small sample size, AICc) model selection to compare models with increasing order polynomials (i.e. linear, quadratic, or cubic trends; see supplementary information for each analysis). The AICc gives a measure of the goodness-of-fit of each potential model while penalising complexity, thereby avoiding overfitting [59]. For each analysis, the model with the lowest AICc, corresponding to the best-fit, was compared to our *a priori* prediction (Fig. 2). The regression models visualised in Figs 3–5 were created using the best-fit models. The difference in AICc between each model and the selected model (ΔAICc), and the Akaike’s weights (ω_i_), were calculated for each model [60]. The Akaike’s weights are interpreted as the probability that each model is the best model of the set [61]. Thus, we can be confident that the functional form (i.e. linear, quadratic, or cubic) of the selected models with high Akaike’s weights best describe the symbiont density trends. The length of the hatchet wing cell (all flies) and stage of pregnancy (females only) were included as fixed effects in all relevant models. Model assumptions were verified using the “performance” package [62]. We analysed the ratio of *Sodalis* to *Wigglesworthia* genomes using the same method (Figs S1–S2).

The effects of rearing hosts on blood diluted with saline and blood enriched with yeast extract were analysed separately (Study 3). To analyse the effect of blood content in diet, we calculated the percentage of defibrinated blood in each treatment for use as a continuous predictor. The same group of flies receiving standard horse blood (i.e. 100% defibrinated blood and 0% yeast extract) were used in both analyses as controls. Due to very high tsetse mortality rates when fed blood containing 5% yeast extract (resulting in a small sample size, Fig. S3), we excluded this group from the analysis. The effects of blood content and blood enrichment on host reproduction were found using the same model selection methodology as above. The most diluted blood treatment (1:3 v/v blood:saline) and most enriched (5% yeast extract) were excluded from the analyses due to the very low reproductive output of hosts reared on these extreme treatments (two and one pupae, respectively).

## Results

### Study 1: symbiont density dynamics across female age

Across the range of adult female ages, the density of *Wigglesworthia* was generally greater than that of *Sodalis* (Fig. 3A and B). *Wigglesworthia* proliferated in early adulthood, up to ~17 days postemergence, after which the density of this symbiont plateaued and declined (Fig. 3A). Accordingly, we found evidence of a negative quadratic effect of host age on *Wigglesworthia* density, as determined by the best-fit model carrying 75% of the AIC weight (i.e. ω_i_ = 0.75; Table S4). For *Sodalis*, after an initial increase in hosts up to 17 days postemergence, the density declined; however, this decline did not continue throughout adulthood and increased slightly in hosts from the age of ~67 days old (Fig. 3B). The model which included the cubic effect of host age was selected as the best-fit for predicting the dynamics of *Sodalis* density and carried 97% of the AIC weight (Table S4). Thus, we observe a divergence in the dynamics of symbiont density for *Wigglesworthia* and *Sodalis* in old hosts, as predicted (Fig. 1A).

### Study 2: symbiont density depending on hunger levels in males

We found no evidence that the density of either *Wigglesworthia* or *Sodalis* varied depending on time since feeding, indicated by selection of the intercept-only model as the best-fit for both symbionts (Fig. 3C and D; Table S5). Thus, we cannot conclude that the density of either symbiont changes throughout the hunger cycle.

### Study 3: effect of dietary manipulation on symbiont density and host reproduction in females

The model including a positive quadratic effect of blood content in diet on *Wigglesworthia* density was determined to be the best-fit, indicating that the density initially decreases with blood dilution and then increases again in hosts receiving very diluted blood (Fig. 4A); however, this model only carried 42% of the AIC weight (Table S6). The intercept-only model carried 36% of the AIC weight, indicating that the evidence for an effect of blood content on symbiont density is very weak. Similarly, we found no evidence that enrichment of blood with yeast extract affects the density of *Wigglesworthia* (Fig. 4B), given by selection of the intercept-only model carrying 71% of the AIC weight (Table S6). We suggest, therefore, that contrary to our predictions (Fig. 1C and D), the nutritional content of the host blood diet does not affect the density of *Wigglesworthia*.

The density of *Sodalis* appeared to decrease slightly with increasingly diluted diets (Fig. 4C). Collectively, the linear, quadratic, and cubic models carried >99.9% of the AIC weight, indicating that there is strong evidence that blood content affects the density of *Sodalis*. The density of *Sodalis* appeared to increase substantially when yeast extract was added to the host diet, although this appeared to plateau with increasing enrichment (Fig. 4D). Accordingly, we found strong evidence of a cubic effect of blood enrichment on the density of *Sodalis*, given by selection of the cubic model carrying >99% of the AIC weight (Table S6). The effect of blood dilution and enrichment on *Sodalis* density correspond well to our directional predictions (Fig. 1C and D).

Both dilution of blood with saline and enrichment with yeast extract appeared to negatively impact female reproduction in terms of reducing pupae weight and the survival time of adult offspring upon emergence (Fig. 5). The detrimental effect of blood dilution on pupae weight appeared to increase with very low blood content in the host diet (Fig. 5A), which is moderately supported by selection of the model including a negative quadratic effect of blood content, carrying 67% of the AIC weight (Table S7). Conversely, we found that pupae weight decreased with addition of any yeast extract to host diet but plateaued with increasing enrichment (Fig. 5B). We found moderate evidence for this nonlinear effect, given by selection of the model including a positive quadratic term, carrying 64% of the AIC weight (Table S7). We found strong evidence that both blood dilution (Fig. 5C) and blood enrichment (Fig. 5D) reduced the time taken for adult offspring to starve upon emergence, as both intercept-only models carried <0.01% of the AIC weight (Table S7).

## Discussion

Here, we investigated the different regulatory forces on the density of *Wigglesworthia* and *Sodalis*, obligate and facultative symbionts of tsetse respectively, within the abdominal tissues of tsetse (which included the digestive tract, reproductive tissues, fat bodies, and Malpighian tubules). By measuring the density of *Wigglesworthia* and *Sodalis* genomes in several ecological scenarios, we aimed to test predictions of how symbiont density would change with host requirements (e.g. across age, over the hunger cycle and depending on the diet). Moreover, we were interested in whether any patterns differed between the two types of symbionts.

First, we measured symbiont density across females in different age groups. We found that both *Wigglesworthia* and *Sodalis* proliferate following emergence (Fig. 3A and B), which supports previous findings [17, 47]. Tsetse generally produce their first offspring around the time that symbionts reach a stable density (~18–23 days [63, 64]). Females may initially invest a large number of resources into growing the population of *Wigglesworthia* until it is sufficient to meet the B-vitamin demands of producing offspring. Upon the onset of reproduction, limiting the resources made available to *Wigglesworthia* and transmission of *Wigglesworthia* to offspring at regular intervals may prevent the continued growth of the *Wigglesworthia* population [65]. Maturation of the immune system in teneral adults is known to leave them susceptible to infection [48], and this may also give an opportunity for symbiont proliferation during this period. Compartmentalisation of symbionts to bacteriomes serves to protect symbionts from immune effectors circulating in the haemolymph [66], so a weakened immune function of the host may have little effect on the primary population of *Wigglesworthia* density. It is possible, however, that regulation via the immune system is a mechanism for hosts to limit *Sodalis* density. The concordant proliferation of *Wigglesworthia* and *Sodalis*, which plateaus at similar times (~17 days; Fig. 3A and B), emphasises the between-symbiont interactions that influences *Sodalis* density. Where dependency on nutrients (e.g. thiamine [40]) produced by the obligate symbiont limits the proliferation of the facultative symbiont, the host may not need to invest greatly in controlling the abundance of *Sodalis* itself.

We hypothesised that due to constant reproduction throughout adult development in female tsetse, the demands for B vitamins would be constant and therefore the density of *Wigglesworthia* would be maintained at a constant level. We found that after the initial proliferation, both *Wigglesworthia* and *Sodalis* appeared to decrease in density in digestive and reproductive tissues (Fig. 3A and B), contrary to our predictions (Fig. 1A). Previous investigations of symbiont abundance in mated adult female tsetse found no positive or negative trend in *Wigglesworthia* during adulthood [17, 47]; however, these findings were based on females of up to 8 weeks (56 days) old, and our observed decrease in *Wigglesworthia* is most noticeable in flies older than 8 weeks (Fig. 3A). Studies of the aphid-*Buchnera* system, a nutritional symbiosis analogous to tsetse-*Wigglesworthia*, observed degradation of the bacteriocytes (the cells which contain the symbiotic bacteria and form the bacteriome) in senescent hosts [67], and that the number of genomes per *Buchnera* cell decreased in old hosts [68]. Additionally, the ploidy of bacteriocytes has been demonstrated to increase in old aphid hosts [69]. Similar effects of ageing in tsetse may contribute to the observed decline in *Wigglesworthia* density (Fig. 3A). Previous studies quantifying *Wigglesworthia* in adult males have revealed a decrease in abundance during the first 8 weeks of adulthood [17, 47], possibly suggesting that the degenerative effect of host ageing on the symbiosis occurs sooner in male hosts than their female counterparts. Studies on the *Sitophilus* weevil have indicated that there may be a benefit to the breakdown of their obligate symbiosis in the somatic tissues, as resources sequestered in the symbiont population can be returned to the host [16]. Such symbiont recycling can help mitigate costs associated with the symbiosis where the requirements of the host change throughout development. However, the weevil system differs to tsetse in that the *Sitophilus* obligate symbiont primarily promotes development of the beetle cuticle and is not required throughout adulthood [70]. As tsetse require *Wigglesworthia* to reproduce but demonstrate reproductive senescence with increasing maternal age [51, 71], the selection pressure on tsetse to maintain or recycle *Wigglesworthia* during old age is likely to be minimal.

We proposed that senescence of the immune system may allow the population of *Sodalis* to increase again in very old flies (Fig. 1A), which we observed in females from the age of ~67 days old (Fig. 3B). In contrast to obligate symbionts, little is known of the mechanisms by which hosts control the abundance of facultative symbionts. Studies on the *Riptortus* bean bug have revealed that the host uses antimicrobial peptides (AMPs) to regulate the abundance of the facultative *Burkholderia* [72]; however, the interactions between *Sodalis* and the tsetse immune system appear complex. Trappeniers and colleagues [73] reported a reduced immune response to *Sodalis*, potentially allowing its colonisation throughout the host lifetime; however, Weiss and his colleagues [74] reported that recognition of *Sodalis* surface proteins by the host induce the expression of immunity related genes. Additionally, *Sodalis* have demonstrated resistance to multiple host AMPs [75–77], and this symbiont may be able to evade host immune effectors by invading host cells [78]. It is likely that the immune system is finely tuned to the presence of *Sodalis* to limit its proliferation without removing it completely [29]. An alternative explanation is that *Sodalis* growth is self-regulated. *Sodalis* has demonstrated the ability to suppress virulence in tsetse via quorum sensing [79], which potentially limits the burden of this symbiont on its host. It might be beneficial for the symbiont to limit its own proliferation during early adulthood to allow host investment in reproduction, as the fitness interests of *Sodalis* and the host are aligned while the host is reproducing due to vertical symbiont transmission [80]. However, exploitation of host resources and unregulated symbiont growth could increase *Sodalis* fitness in hosts that are no longer reproducing as increased symbiont density may promote the likelihood of horizontal transmission. The mechanism by which *Sodalis* invades host cells indicates that it evolved from parasitic ancestors [81], and it is plausible that a facultative symbiont, which has recently transitioned to host-associated living, may express selfish and opportunistic traits characteristic of parasitism under certain contexts. Indeed, *Sodalis* spp. have the capacity to colonise a broad range of host tissues [82]. As *Sodalis* potentially confers a benefit to the host [42], this symbiont may proliferate in an attempt to boost fecundity in late-aged females, which would also promote *Sodalis* fitness via vertical transmission. Further studies that measure how the density of *Sodalis* responds to the knockout of candidate regulatory mechanisms in both tsetse and *Sodalis* could be used to test whether *Sodalis* density is regulated by the host immune system or if proliferation of this symbiont is self-regulated.

We observed that the density of *Wigglesworthia* is generally greater than that of *Sodalis* within the digestive and reproductive tissues (Fig. 3). This was consistent among our three studies, which tested both males and females of a range of ages (Figs 3 and 4), and is consistent with previous studies which measured symbiont densities within whole flies [17, 47]. It is potentially surprising given that *Sodalis* demonstrates a wider tissue tropism than *Wigglesworthia* and does not rely on the host to mediate cell replication [37]. As our primer pairs were designed to target single-copy genes in both symbionts and tsetse, it is possible that *Wigglesworthia* demonstrates a high prevalence of polyploidy which *Sodalis* does not or that the host can direct a large number of resources into the relevant tissues for the maintenance of *Wigglesworthia.*

Due to its presence in the midgut, we hypothesised that *Sodalis* would proliferate following a blood meal; however, we found no evidence of changes in the density of either *Wigglesworthia* or *Sodalis* throughout the hunger cycle (Fig. 3C and D). Proliferation of midgut bacteria following a blood meal is demonstrated in other blood-feeding insects, such as mosquitoes, peaking at 24–36 hours postblood feed [83]. We consider several explanations for why we did not observe a similar proliferation by *Sodalis*. First, the gut symbionts of adult mosquitoes are acquired environmentally, and although *Sodalis* is a facultative symbiont we consider that there is still an evolutionary history which may subject *Sodalis* to a greater degree of regulation—whether host- or symbiont-mediated—than transient symbionts of mosquitoes. Second, mosquitoes possess very diverse gut microbiomes [84], and it may be that only specific bacteria demonstrate proliferation with blood intake. Third, the density of microbiota within tsetse is relatively low [85], rending proliferation potentially challenging to detect, particularly if large variation in symbiont density exists between individuals. Finally, the growth of cultured *Sodalis* has been shown to be slow relative to other bacteria, with a doubling time of ~26 hours [86]. The growth kinetics *in vitro* may correspond to the within-host growth of *Sodalis*, in which case density fluctuation within a hunger cycle is likely to be weak.

By rearing tsetse on different diets, we aimed to manipulate the requirements of hosts for symbiont-derived micronutrients. We hypothesised that hosts would regulate the abundance of *Wigglesworthia* to maximise the benefit of hosting this symbiont population, whereas the growth of *Sodalis* would respond directly to the availability of nutrients in the diet (Fig. 1C). We did not find strong evidence of the density of *Wigglesworthia* changing in hosts reared on different diets (Fig. 4A and B). We consider two explanations. First, it is possible that the cost of maintaining *Wigglesworthia* is relatively low, such that even on a diet with very little macronutrient content, there is still a net benefit to hosting a large *Wigglesworthia* density. Second, the cost of reducing *Wigglesworthia* may be substantial, and it may not be beneficial to actively regulate according to host nutrition. The intracellular localisation of *Wigglesworthia* support the second suggestion, as reduction in the cell density of *Wigglesworthia* may require autophagy of the host’s own bacteriocytes [16, 29, 67]. Aphids are reported to harbour different densities of their obligate symbiont (which resides within their bacteriome) according to their host plant [13]. In contrast to aphids which feed on one plant, however, tsetse do not feed from one host individual but find a new blood meal every few days and may be able to compensate for poor diet via foraging. Tsetse may therefore regulate *Wigglesworthia* according to long-term nutritional requirements rather than short-term fluctuations in diet. The stationary dynamics of *Wigglesworthia* density observed throughout the hunger cycle (Fig. 3C) provides additional support to the fact that tsetse may maintain a constant, optimal density of *Wigglesworthia*. When the relative cost of supporting *Wigglesworthia* is great (such as when dietary energy is limited) or where the requirements for *Wigglesworthia* are reduced (such as when the host receives a higher quality diet) it is possible that tsetse reduce *Wigglesworthia* metabolism as an alternative to reducing cell density [11, 87]. In future studies, using reverse transcription qPCR to measure gene expression in *Wigglesworthia* could test if B vitamin synthesis by this symbiont is regulated according to the nutritional requirements of the host.

In contrast to *Wigglesworthia*, we found that the density of *Sodalis* changed with dietary manipulation as predicted (Fig. 1C), whereby the density of *Sodalis* was lower in hosts reared on diets with lower blood content (Fig. 4C), and greater in hosts reared on diets enriched with yeast, an exogenous source of B vitamins (Fig. 4D). Reduced macronutrient availability, as well as decreased thiamine production by *Wigglesworthia* [40], in tsetse receiving diets of diluted blood may limit the growth of *Sodalis*. As there is no clear benefit of hosting a larger population of *Sodalis* when tsetse are supplied with a higher quality diet, we infer that the resulting increase in *Sodalis* density in hosts receiving blood enriched with yeast extract is not the result of tsetse regulating *Sodalis* to a higher density, rather that this is mediated by *Sodalis* itself. Rearing tsetse on a diet of diluted blood imposed detrimental effects on host reproduction, indicated by the reduced pupae weight and starvation time of offspring upon emergence (Fig. 5A and C), which is consistent with previous findings [51]. Enriching blood with yeast extract also impacted reproduction (Fig. 5B and D) and severely reduced survival in hosts administered 5% yeast extract (Fig. S3), possibly indicating a toxic effect of this treatment. A reduction in tsetse fecundity has previously been observed with supplementation of host diet with yeast extract [50]. Hosts experiencing nutritional stress possibly also suffer from impaired immunity [88]. As well as being provided with a greater complement of nutrients, the host may have a reduced ability to limit *Sodalis* proliferation, hence the increased density of *Sodalis* (Fig. 5D). Any detrimental effects on immunity caused by the dietary treatments, however, do not appear to affect the density of *Wigglesworthia* (Fig. 4A and B).

## Conclusion

We found evidence that the ecological contexts that influence symbiont growth and density in tsetse are different for *Wigglesworthia* and *Sodalis*, their obligate and facultative symbionts, respectively. Although we observed a decline in *Wigglesworthia* density in older flies, we found no evidence that *Wigglesworthia* density is actively adjusted to meet short-term nutritional requirements; rather, we suggest that it is maintained at a high abundance by the host to maximise long-term benefits. Variation in nutrient availability appears to have little impact on the density of *Wigglesworthia*, supporting the hypothesis that density is primarily controlled by the host. *Sodalis*, however, appears to respond dynamically to the conditions of its environment, and factors such as host age and nutrient availability have significant impacts on the density of this symbiont. We suggest that due to the recent transition to host-associated living, both the host and symbiont possess a degree of control over the density of this facultative symbiont, potentially via host immune function and self-regulation. The potential for vertically transmitted, facultative symbionts to transition along the mutualism–parasitism spectrum according to the ecological context, and the evolutionary outcomes of such interactions, warrant further investigation.

## Supplementary Material

ISME_Com_supporting_info_final_submission_ycaf108

## Data Availability

The dataset and code generated during the current study are available in the Dryad repository (https://doi.org/10.5061/dryad.0gb5mkm9f).
