# Supplementary material for: How do host age and nutrition affect density regulation of obligate versus facultative bacterial symbionts? Insights from the tsetse fly
Source: ISME Commun. 2025 Jun 27;5(1):ycaf108. doi: 10.1093/ismeco/ycaf108 (PMC12309370; doi:10.1093/ismeco/ycaf108)
Supplement: ISME_Com_supporting_info_final_submission_ycaf108 [file isme_com_supporting_info_final_submission_ycaf108.docx]

**Supplementary material: How do host age and nutrition affect density regulation of obligate versus facultative bacterial symbionts? Insights from the tsetse fly**

Mathilda Whittle^1,2^*, Antoine M.G. Barreaux^3,4^, Lee R. Haines^5^, Michael B. Bonsall^6,7^, Sinead English^1Ϯ^ and Fleur Ponton^2 Ϯ^

^1^Faculty of Life Sciences, University of Bristol, Bristol, UK; ^2^School of Natural Sciences, Macquarie University, NSW, Australia; ^3^UMR INTERTRYP, CIRAD, Montpellier, France; ^4^Animal Health Theme, ICIPE, Nairobi, Kenya; ^5^Department of Biological Sciences, University of Notre Dame, Indiana, USA; ^6^Department of Biology, University of Oxford, Oxford, UK; ^7^St Peter’s College, Oxford, UK.

**Tsetse feeding**

We supplied bloodmeals to tsetse by pooling 25 mL blood on metal trays (47cm x 40cm) and covering with a silicone membrane, taking care to remove air bubbles between the sheet and membrane. Plates were then warmed to 36°C using heating mats, and cages were placed on top to allow flies to feed *ad libitum* until visibly engorged (approx. 15 mins). Membranes and metal trays were sterilised at 110°C overnight between feeds. For flies used in study 2 and 3, we added ATP to stimulate feeding (200μL per 25 mL blood), which was made by diluting 5.51 g of adenosine 5’-triphosphate disodium per 100 ml of Reverse Osmosis water.

**Study 1: symbiont density dynamics across female age**

For investigating symbiont density dynamics across adult age, we used females from 16 different age groups spanning the age range of the tsetse colony; from approximately 6 hours to 88 days post emergence, with n = 10 for each group. Flies were killed by freezing at -80 °C, 48 hours following their final blood meal (excluding newly emerged flies, which were unfed) and processed for qPCR quantification of symbiont density (see below). Upon maturation, female tsetse produce offspring at regular intervals (every 9-10 days) throughout adulthood. Within individuals, the density of *Wigglesworthia* and *Sodalis* is likely to fluctuate during adulthood due to transmission of both symbionts to developing larvae (51), however, we anticipated that these short-term fluctuations were not likely to be observed at a population level due to the reproductive cycles being unaligned between females.

**Study 2: symbiont density depending on hunger levels in males**

We reared newly emerged males for approximately 4 weeks on a standard horse blood diet. We anticipated that by using males, small changes in symbiont density across the hunger cycle may be observed, as any contribution of fluctuating symbiont populations in the female milk glands are eliminated. We caged males without females to prevent any reduction in *Sodalis* density due to mating (37). To maximise likelihood of feeding and to allow complete digestion of the previous meal, flies were starved for 4 days prior to the final meal (at 4 weeks old). All flies appeared fully engorged at the final meal. Flies were killed by freezing at -80 °C at 6 (n = 13), 24 (n = 14), 48 (n = 12) or 96 (n = 13) hours following the final meal and processed for qPCR quantification of symbiont density.

**Study 3: effect of dietary manipulation on symbiont density in females**

Adult females were caged upon emergence at a female: male ratio of 4:1. We reared flies for 6 weeks on one of 10 dietary treatments (table 1). We used normal saline (0.9% NaCl (w/v) in water) to reduce the nutritional content of the diet at varying ratios, and yeast extract to fortify the diet with B vitamins at increasing concentrations. For addition to the blood diet, we dissolved 0.4, 0.8, 1.6 and 4 g of dried yeast extract (Merck, cat. No. 70161) in aliquots of 10 mL reverse osmosis (RO) water, which we then sterilised using syringe filters (0.2 μm). The yeast solutions were then added to aliquots of 70 mL blood to create blood enriched with 0.5, 1, 2 and 5% w/v yeast extract. Diets were pre-prepared with fresh blood and stored for up to two weeks at 3°C. We assumed that increased blood intake could not compensate for blood dilution in flies receiving these treatments, as previous studies have demonstrated that reduced red blood cell:serum ratio impairs reproductive output (1). To ascertain any detrimental effects of the treatments on host survival, we counted the number of living females daily. Pupae were collected from each group and incubated under the same environmental conditions until emergence. We recorded the weight at deposition of pupae produced by each treatment group, and the number of days for the unfed adult offspring to die post emergence, to determine if the dietary treatments affected host reproduction and resource provisioning to offspring. All surviving females were killed by freezing at -80°C, 48 hours after their final blood meal, and processed for qPCR quantification of symbiont density.

Sample preparation and DNA extraction method

The total content of the abdomen was removed by dissection into 180 μL ATL buffer using the DNeasy blood and tissue kit (Qiagen, CA). Instruments and surfaces were washed with 70% ethanol to prevent cross-contamination. Tissue in ATL buffer was transferred to a microcentrifuge tube and centrifuged at 6000 g for 1 min to pellet tissue. 20 μL proteinase K was added then briefly vortexed. Samples were incubated at 56°C for 2 hours, vortexing every 30 mins for 15 s. 200 μL Al buffer was added, then vortexed. 200 μL ice-cold ethanol (96 – 100%) added, then vortexted. Samples were centrifuged at 14,000 g for 3 mins to pellet any remaining tissue. Supernatant was transferred into a spin-column placed inside a collection tube, the centrifuged at 6000 g for 1 min. The flow-through was discarded alongside the collection tube. The spin column was placed in a new collection tube and 500 μL AW1 buffer added, then re-centrifuged. Spin column placed in new collection tube and 500 μL AW2 buffer added. Samples incubated at room temperature for 5 mins, the centrifuged at 14,000 g for 3 mins to dry DNA membrane. Spin column placed into new microcentrifuge tube, then 100 μL AE buffer added and incubated at room temperature for 1 min. Samples centrifuged at 6000 g for 1 min to elute DNA. Another 100 μL AE buffer added to spin column, incubated for 1 min and centrifuged. Spin column discarded. Extracted DNA was desiccated for short-term storage at -30°C using an Eppendorf Vacufuge plus (Eppendorf, UK). Dried DNA was then rehydrated with 50 µL PCR grade water (Fisher Biotec, Australia) for analysis.

PCR amplification

We used non-experimental flies to obtain PCR products by amplification of alpha-tubulin, thiC and exochitinase genes. PCR amplification was performed using a Mastercycler X50s (Eppendorf, Hamburg). Amplifications were obtained using GoTaq colorless master mix (Promega, WI) in a final volume of 25 µL (including 5 µL of template). The cycling conditions for PCR amplification were: 10 min./95°C, 25 cycles (30 sec./95°C, 45 sec./Ta and 1 min./72°C). The cycling conditions for qPCR amplification were: 5 min./95°C, 40 cycles (10 sec./95°C, 20 sec./Ta and 30 sec./72°C). Ta for each pair of primers is given in table S1. We then purified the PCR products using QIAquick PCR purification kit (Qiagen, CA). DNA concentration was measured using a Qubit 4 fluorometer (Invitrogen, MA).

qPCR Assay optimisation and standard curves

To determine the linear dynamic range for each target gene, we constructed standard curves using four-fold serial dilutions (over nine points) of PCR products of known concentration (concentration ranges given in table S2). We verified the efficiency of amplification and dynamic range for purified PCR products (91.8-97.0%) against standard curves constructed from total DNA from nonexperimental flies (95.4-98.3%). We confirmed the specificity of primer annealing by performing a melt-curve analysis. Amplifications were obtained using PowerUP SYBR Green master mix (Applied Biosystems, MA) in a final volume of 10 µL, including 4 µL of template. We found optimal primer concentrations by running titrations between 250 nM and 900 nM of the Forward and Reverse primers. Primer concentration of 500 nM was chosen as it yielded the lowest quantification cycle (Cq) for all primer pairs. We diluted the DNA samples with PCR grade water (Fisher Biotec, Australia) to fit within the dynamic range of the standard curves (table S2).

Study 1: symbiont density dynamics across female age

To compare the dynamics of *Wigglesworthia* and *Sodalis* across female age, we fit models including intercept-only, linear, quadratic and cubic effects of host age (in days), then selected the model with the lowest AICc for each symbiont.

**Study 2: symbiont density depending on hunger levels in males**

To compare the dynamics of *Wigglesworthia* and *Sodalis* throughout the hunger cycle, we fit models including intercept-only, linear, quadratic and cubic effects of the amount of time since feeding (in hours), then selected the model with the lowest AICc for each symbiont.

**Study 3: effect of dietary manipulation on symbiont density in females**

The effect of blood dilution on *Wigglesworthia* and *Sodalis* densities was investigated by fitting models including intercept-only, linear, quadratic and cubic effects of the amount of defibrinated blood in host diet (%), then the model with the lowest AICc was selected for each symbiont. Likewise, the effect of blood enrichment on *Wigglesworthia* and *Sodalis* densities was investigated by fitting models including intercept-only, linear, quadratic and cubic effects of the amount of yeast extract in host diet (% w/v), inputted as a continuous variable.

Table S1 Primers for PCR and qPCR.

| Target species | Target gene | Amplicon size (bp) | Forward primer sequence | Backward primer sequence | Annealing temperature / T_a_ (°C) |
| --- | --- | --- | --- | --- | --- |
| *Glossina morsitans morsitans* | *α-tubulin* | 160 | CAA-GGA-GGA-CGC-TGC-GAA-TA | CCA-CCA-CCG-AAC-GAA-TGG-AA | 62.4 |
| *Wigglesworthia glossinidia* | Thiamine biosynthesis protein (*thiC*) | 130 | AAG-TTA-TGA-TAG-AAG-GAC-CAG-GAC | CCC-GGA-GCA-ATA-TCA-GTA-GTT-AG | 55.3 |
| *Sodalis glossinidius* | *Exochitinase* | 120 | TGG-GGA-CAG-TAC-GAT-GGC-AGA-GC | TCA-TAG-GCG-GTC-GGG-GAT-AAT-TGC-G | 68.3 |

Table S2 Concentration range used to create standard curves and dilution factor for DNA samples for each target gene.

| Target species | Amplicon concentration range for standard curve (ng/µL) | Sample dilution factor |
| --- | --- | --- |
| *Glossina morsitans morsitans* | 3.00x10^-9^–4.86x10^-5^ | 0.25^3^ |
| *Wigglesworthia* glossinidia | 2.16x10^-9^–3.54x10^-5^ | 0.25^6^ |
| *Sodalis glossinidius* | 1.28x10^-9^–2.09x10^-5^ | 0.25^4^ |

Table S3 Scale of inference and sample sizes for statistical analysis.

| Scale of inference | Scale at which the factor of interest is applied | Number of replicates at the appropriate scale |
| --- | --- | --- |
| Individual | Individual (16 age groups) | 9 – 10 flies per age group |
|  | Individual (4 time points) | 12 – 14 flies per time point |
|  | Individual (9 dietary treatments) | 8 – 13 flies per treatment |

Table S4 Model comparison used to predict symbiont density throughout development of adult female tsetse. WL denotes the length of the hatchet wing cell, and P the stage of pregnancy. Models in bold were used to produce plots. k indicates the number of parameters.

| Response | Effects | k | AICc | ΔAICc | ω_i_ |
| --- | --- | --- | --- | --- | --- |
| log(*Wigglesworthia* genomes/*Glossina* genomes) | **Intercept + WL + P + Age + Age^2^** | **8** | **348.97** | **0** | **0.75** |
|  | Intercept + WL + P + Age + Age^2^ + Age^3^ | 9 | 351.22 | 2.24 | 0.25 |
|  | Intercept + WL + P + Age | 7 | 360.45 | 11.47 | <0.01 |
|  | Intercept + WL + P | 6 | 380.95 | 31.97 | <0.01 |
| log(*Sodalis* genomes/*Glossina* genomes) | **Intercept + WL + P + Age + Age^2^ + Age^3^** | **9** | **301.78** | **0** | **0.97** |
|  | Intercept + WL + P | 6 | 310.10 | 8.32 | 0.02 |
|  | Intercept + WL + P + Age | 7 | 311.23 | 9.44 | <0.01 |
|  | Intercept + WL + P + Age + Age^2^ | 8 | 313.36 | 11.58 | <0.01 |
| log(*Sodalis* genomes/*Wigglesworthia* genomes) | **Intercept + WL + P + Age + Age^2^ + Age^3^** | **9** | **389.83** | **0** | **0.96** |
|  | Intercept + WL + P + Age + Age^2^ | 8 | 396.09 | 6.26 | 0.04 |
|  | Intercept + WL + P + Age | 7 | 402.72 | 12.89 | <0.01 |
|  | Intercept + WL + P | 6 | 412.59 | 22.76 | <0.01 |

Table S5 Model comparison used to predict symbiont density across the hunger cycle for adult male tsetse. WL denotes the length of the hatchet wing cell. Models in bold were used to produce plots. k indicates the number of parameters.

| Response | Effects | k | AICc | ΔAICc | ω_i_ |
| --- | --- | --- | --- | --- | --- |
| log(*Wigglesworthia* genomes/*Glossina* genomes) | **Intercept + WL** | **3** | **146.44** | **0** | **0.49** |
|  | Intercept + WL + Time_since_meal | 4 | 147.66 | 1.22 | 0.26 |
|  | Intercept + WL + Time_since_meal^2^ | 5 | 149.02 | 2.58 | 0.13 |
|  | Intercept + WL + Time_since_meal^3^ | 6 | 149.38 | 2.94 | 0.11 |
| log(*Sodalis* genomes/*Glossina* genomes) | **Intercept + WL** | **3** | **120.09** | **0** | **0.46** |
|  | Intercept + WL + Time_since_meal | 4 | 121.23 | 1.14 | 0.26 |
|  | Intercept + WL + Time_since_meal^2^ | 5 | 121.60 | 1.51 | 0.22 |
|  | Intercept + WL + Time_since_meal^3^ | 6 | 124.04 | 3.95 | 0.06 |
| log(*Sodalis* genomes/*Wigglesworthia* genomes) | **Intercept + WL** | **3** | **130.39** | **0** | **0.68** |
|  | Intercept + WL + Time_since_meal | 4 | 132.69 | 2.30 | 0.21 |
|  | Intercept + WL + Time_since_meal^2^ | 5 | 135.16 | 4.77 | 0.06 |
|  | Intercept + WL + Time_since_meal^3^ | 6 | 135.86 | 5.47 | 0.04 |

Table S6 Model comparison used to predict symbiont density in adult female tsetse reared on manipulated diets: blood diluted with saline and blood enriched with yeast extract. WL denotes the length of the hatchet wing cell, and P the stage of pregnancy. Models in bold were used to produce plots. k indicates the number of parameters.

| Response | Effects | k | AICc | ΔAICc | ω_i_ |
| --- | --- | --- | --- | --- | --- |
| **Blood dilution with saline:** |  |  |  |  |  |
| log(*Wigglesworthia* genomes/*Glossina* genomes) | **Intercept + WL + P + Blood_content^2^** | **8** | **148.09** | **0** | **0.42** |
|  | Intercept + WL + P | 6 | 148.41 | 0.32 | 0.36 |
|  | Intercept + WL + P + Blood_content^3^ | 9 | 150.75 | 2.66 | 0.11 |
|  | Intercept + WL + P + Blood_content | 7 | 150.78 | 2.70 | 0.11 |
| log(*Sodalis* genomes/*Glossina* genomes) | **Intercept + WL + P + Blood_content^2^** | **8** | **91.60** | **0** | **0.48** |
|  | Intercept + WL + P + Blood_content | 7 | 92.13 | 0.53 | 0.37 |
|  | Intercept + WL + P + Blood_content^3^ | 9 | 94.00 | 2.40 | 0.15 |
|  | Intercept + WL + P | 6 | 119.58 | 27.98 | <0.01 |
| log(*Sodalis* genomes/*Wigglesworthia* genomes) | **Intercept + WL + P + Blood_content^2^** | **8** | **171.70** | **0** | **0.75** |
|  | Intercept + WL + P + Blood_content^3^ | 9 | 174.31 | 2.61 | 0.20 |
|  | Intercept + WL + P + Blood_content | 7 | 177.34 | 5.64 | 0.04 |
|  | Intercept + WL + P | 6 | 186.51 | 14.81 | <0.01 |
| **Blood enrichment with yeast extract:** | |  |  |  |  |
| log(*Wigglesworthia* genomes/*Glossina* genomes) | **Intercept + WL + P** | **6** | **92.161** | **0** | **0.71** |
|  | Intercept + WL + P + Yeast_content | 7 | 95.032 | 2.87 | 0.17 |
|  | Intercept + WL + P + Yeast_content^3^ | 9 | 96.468 | 4.31 | 0.08 |
|  | Intercept + WL + P + Yeast_content^2^ | 8 | 98.10 | 5.94 | 0.04 |
| log(*Sodalis* genomes/*Glossina* genomes) | **Intercept + WL + P + Yeast_content^3^** | **9** | **65.836** | **0** | **>0.99** |
|  | Intercept + WL + P + Yeast_content^2^ | 8 | 84.038 | 18.20 | <0.01 |
|  | Intercept + WL + P + Yeast_content | 7 | 96.14 | 30.30 | <0.01 |
|  | Intercept + WL + P | 6 | 121.51 | 55.68 | <0.01 |
| log(*Sodalis* genomes/*Wigglesworthia* genomes) | **Intercept + WL + P + Yeast_content^2^** | **8** | **93.57** | **0** | **0.65** |
|  | Intercept + WL + P + Yeast_content^3^ | 9 | 94.85 | 1.28 | 0.34 |
|  | Intercept + WL + P + Yeast_content | 7 | 102.76 | 9.19 | <0.01 |
|  | Intercept + WL + P | 6 | 125.93 | 32.36 | <0.01 |

Table S7 Model comparison used to predict the effect of dietary manipulations on host reproduction. Models in bold were used to produce plots. k indicates the number of parameters.

| Response | Effects | k | | AICc | ΔAIC | ω_i_ |
| --- | --- | --- | --- | --- | --- | --- |
| **Blood dilution with saline:** | |  |  |  |  |  |
| Pupae weight | **Intercept + Blood_content^2^** | **4** | | **575.40** | **0** | **0.67** |
|  | Intercept + Blood_content^3^ | 5 | | 576.85 | 1.45 | 0.32 |
|  | Intercept + Blood_content | 3 | | 583.66 | 8.26 | 0.01 |
|  | Intercept | 2 | | 634.68 | 59.28 | <0.01 |
| Starvation time | **Intercept + Blood_content** | **3** | | **487.35** | **0** | **0.47** |
|  | Intercept + Blood_content^2^ | 4 | | 488.09 | 0.74 | 0.33 |
|  | Intercept + Blood_content^3^ | 5 | | 489.07 | 1.73 | 0.20 |
|  | Intercept | 2 | | 501.70 | 14.35 | <0.01 |
| **Blood enrichment with yeast extract:** | |  | |  |  |  |
| Pupae weight | **Intercept + Yeast_content^2^** | **4** | | **356.05** | **0** | **0.64** |
|  | Intercept + Yeast_content^3^ | 5 | | 357.29 | 1.25 | 0.34 |
|  | Intercept + Yeast_content | 3 | | 362.92 | 6.88 | 0.02 |
|  | Intercept | 2 | | 372.57 | 16.52 | <0.01 |
| Starvation time | **Intercept + Yeast_content** | **3** | | **292.89** | **0** | **0.47** |
|  | Intercept + Yeast_content^3^ | 5 | | 293.42 | 0.53 | 0.36 |
|  | Intercept + Yeast_content^2^ | 4 | | 294.88 | 1.98 | 0.17 |
|  | Intercept | 2 | | 310.18 | 17.29 | <0.01 |


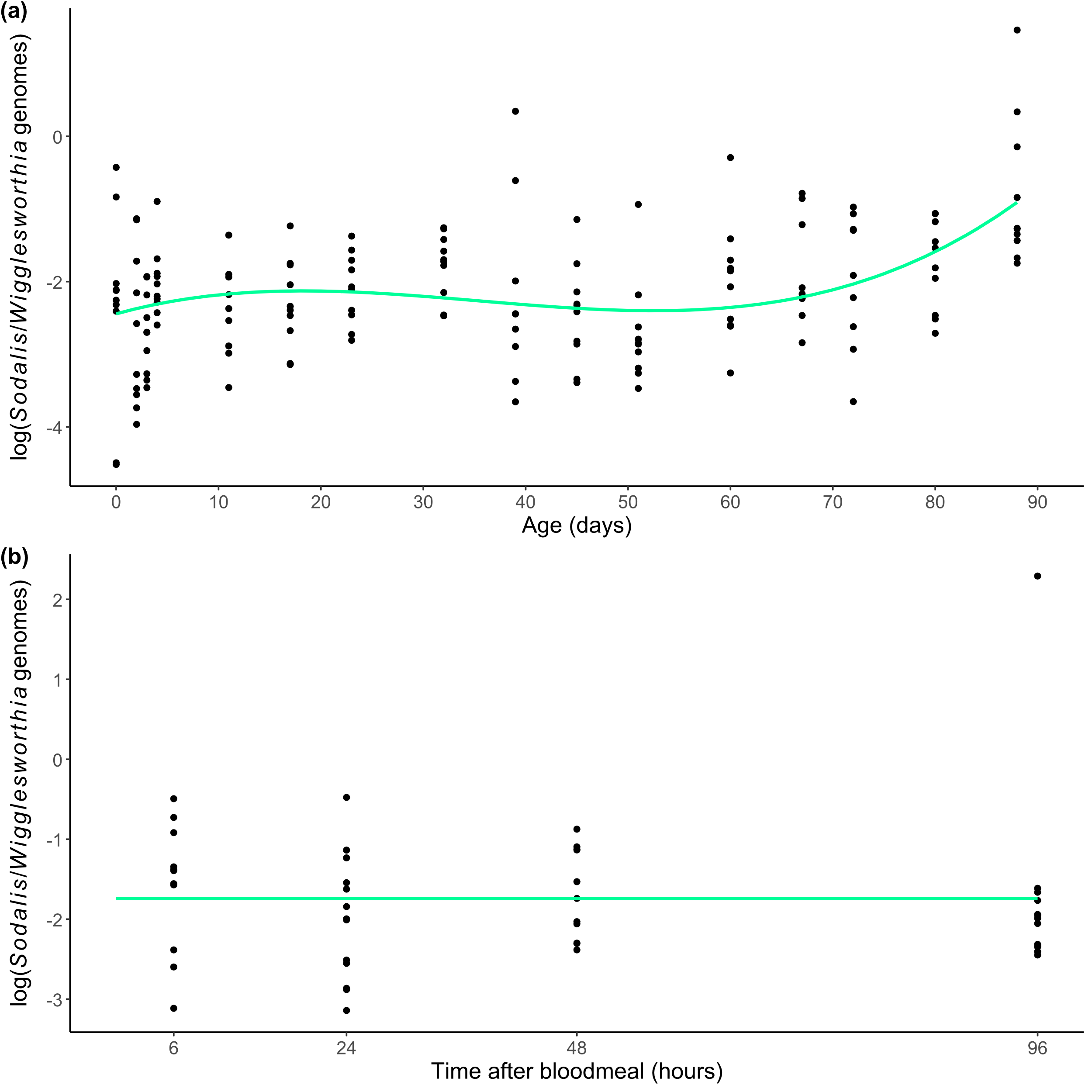


Fig S1 Ratio of *Sodalis* to *Wigglesworthia* genomes (a) throughout adult development (females), and (b) throughout the hunger cycle (4-week old males). Regression lines indicate predictor effects of selected models (tables S3-S4).


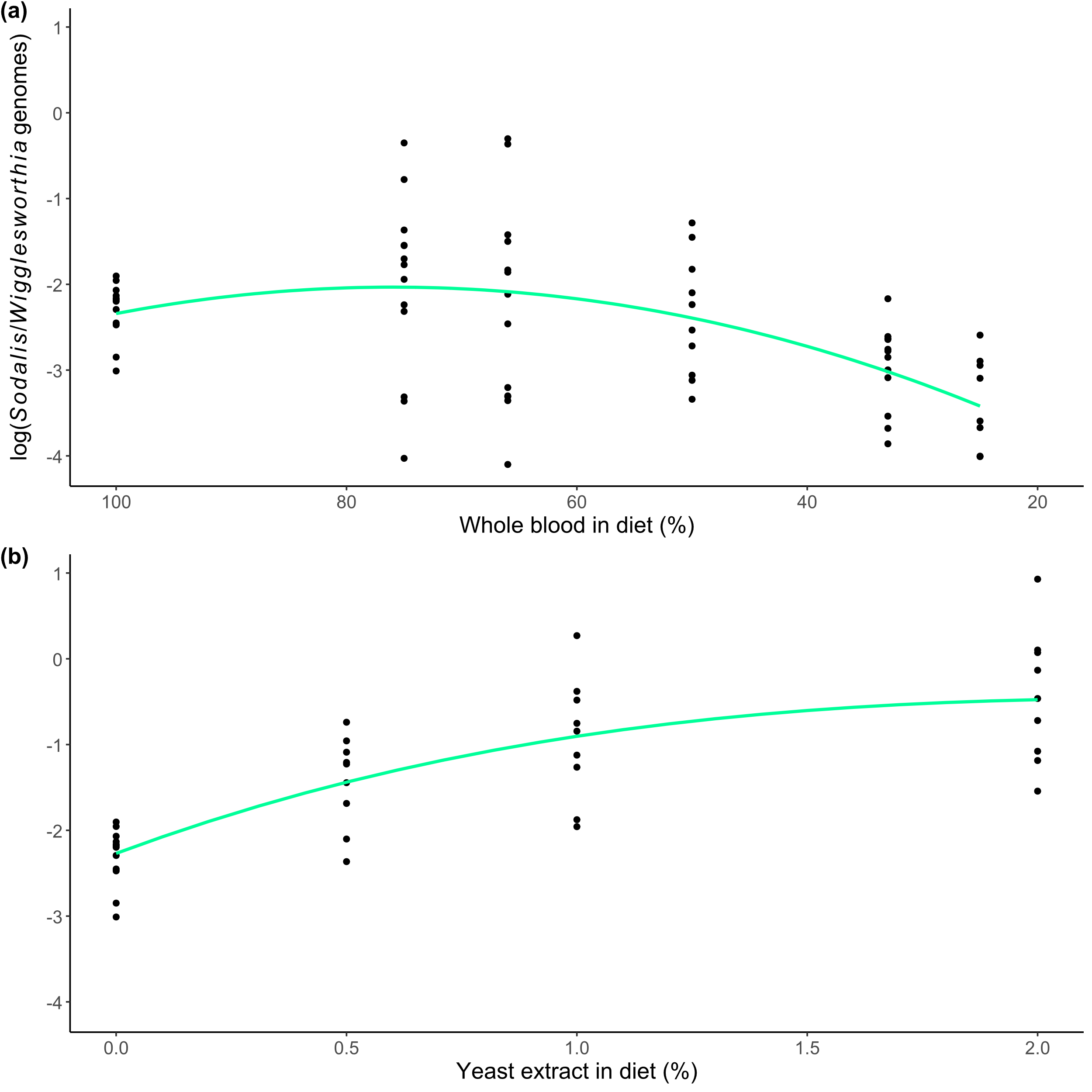


Fig S2 Effect of dietary manipulation on the ratio of *Sodalis* to *Wigglesworthia* genomes in 6-week old female tsetse. (a) according to blood concentration (%). (b) upon yeast extract (% w/v) supplementation. Regression lines indicate predictor effects of selected models (table S6). Note that the y-axis is on different scales between the two symbionts.


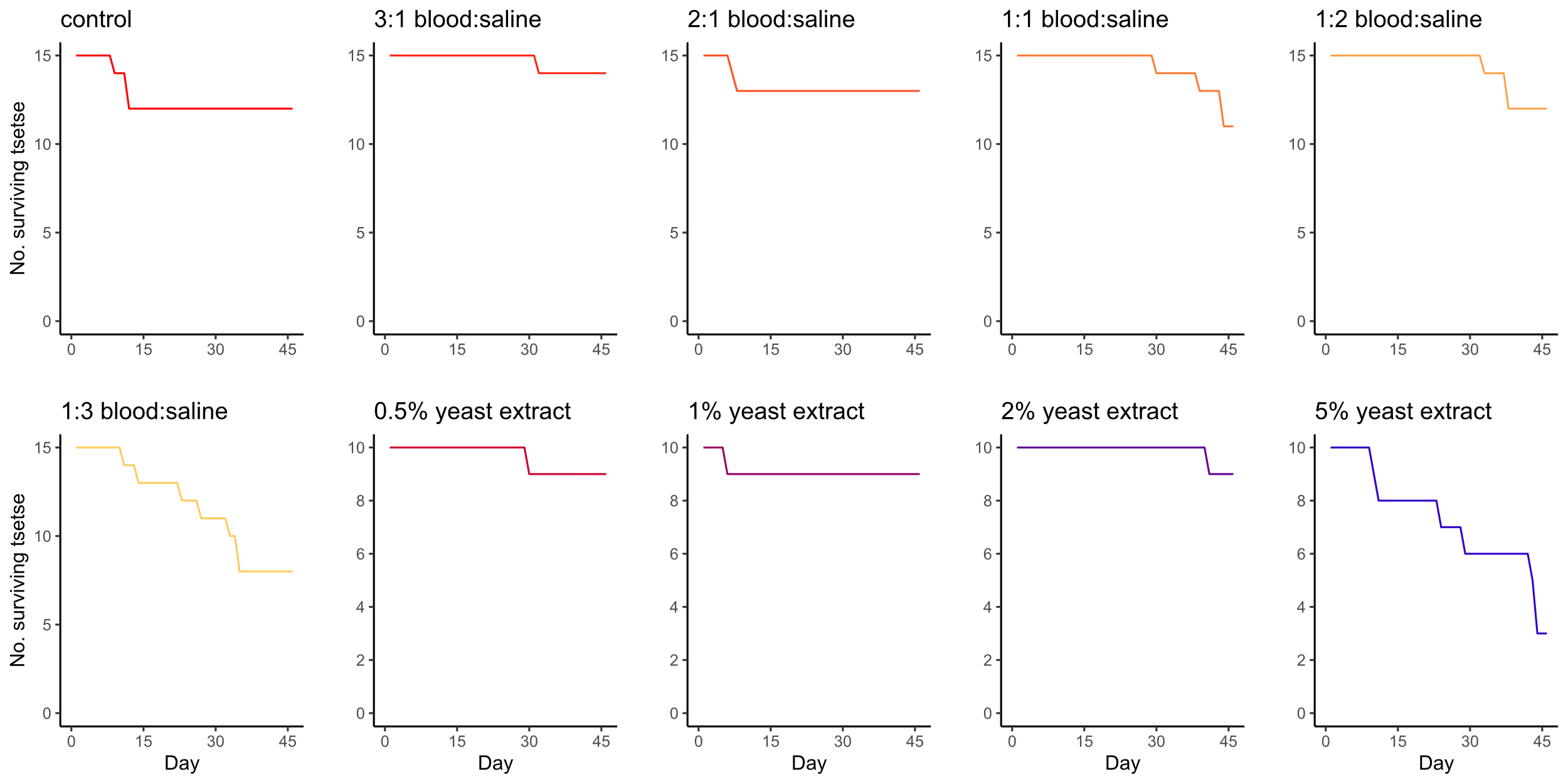


Fig S3 Survival of female tsetse reared for six weeks on dietary treatments. At start of experiment, n = 15 for control and diluted blood treatment groups and n = 10 for yeast-enriched treatment group

**References**

1. Lord JS, Leyland R, Haines LR, Barreaux AMG, Bonsall MB, Torr SJ*, et al.* 2021 Effects of maternal age and stress on offspring quality in a viviparous fly. *Ecol. Lett.* 24, 2113-22.
